# Effect of *Cordyceps* spp. and Cordycepin on Functions of Bones and Teeth and Related Processes: A Review

**DOI:** 10.3390/molecules27238170

**Published:** 2022-11-24

**Authors:** Karol Jędrejko, Katarzyna Kała, Katarzyna Sułkowska-Ziaja, Jolanta Pytko-Polończyk, Bożena Muszyńska

**Affiliations:** 1 Department of Pharmaceutical Botany, Faculty of Pharmacy, Jagiellonian University Medical College, 9 Medyczna Street, 30-688 Kraków, Poland; 2 Chair and Department of Integrated Dentistry, Faculty of Medicine, Jagiellonian University Medical College, 4 Montelupich Street, 31-155 Kraków, Poland

**Keywords:** adenosine, *Cordyceps militaris*, medicinal mushrooms, cordycepin, bone, osteoporosis, skeletal system diseases

## Abstract

*Cordyceps* spp. (belonging to the *Ascomycota* group) are entomopathogenic mushrooms that have traditionally been used in ethnomedicine in Asian countries such as China, Japan, Korea, and India. They are unique parasites of larvae of selected species of moths. *Cordyceps militaris* is one of the best sources of cordycepin. Worldwide, osteoporosis is one of the most common bone diseases, whose pharmacotherapy includes various medical interventions; however, the research and development of new molecules and new drugs is required. The impact of adenosine receptors (ARs) on the purinergic signaling pathway may regulate proliferation, differentiate dental pulp stem cells and bone marrow, and modulate osteogenesis and bone repair. The aim of the review was to collect and analyze the available data on the effects of *Cordyceps* spp. or cordycepin on bone function and related processes. To the best of our knowledge, this is the first systematic review in this perspective, not necessarily using mushroom raw material or even the isolated parent compound cordycepin, but new molecules that are analogs of nucleosides, such as those from *C. militaris.* This review found that *Cordyceps* spp. or isolated cordycepin interacts via the AR, 5′ adenosine monophosphate-activated protein kinase (AMPK), and adenosine-5′-triphosphate (ATP) signaling pathway and evaluated their impact on bones, teeth, and dental pulp. *Cordyceps* spp. was found to have the potential to develop regenerative medicines, thus providing an opportunity to expand the treatment or intervention methods in the recovery after traumatic injuries, convalescence, and terminal-stage or devastating diseases.

## 1. Introduction

Common metabolic bone diseases in humans include the following: osteoporosis, rickets in children, and osteomalacia in adults, followed by fluorosis and primary hyperparathyroidism. Some rare bone diseases include Paget’s disease, bone tumor-induced osteomalacia, osteosarcoma, osteopetrosis (Albers-Schönberg disease), and fibrous dysplasia. Bone homeostasis refers to maintaining the balance between bone resorption and bone formation by osteoclasts and osteoblasts, respectively. Osteoporosis is characterized by a reduction in bone mass and microarchitectural deterioration of bone tissue. The low bone mass, along with increased bone fragility, puts osteoporosis patients at risk of fractures [1,2].

It has been estimated that in the European Union (EU), approximately 22 million women and 5.5 million men suffer from osteoporosis [3]. Worldwide, systematic reviews and meta-analyses have estimated the prevalence of osteoporosis to be 18.3% in the general population (it was 23.1% in women, whereas in men was nearly twice as low at 11.7%) [4].

Pharmacotherapy for osteoporosis includes various medicines such as estrogen, calcitonin, selective estrogen receptor modulators (SERMs) such as raloxifene, bisphosphonates (alendronate, risedronate, ibandronate, and zoledronic acid), human monoclonal antibodies to the receptor activator of the nuclear factor-κB (NF-κB) ligand (RANKL) such as denosumab, and the parathyroid hormone analog teriparatide. Recently, two new drugs—abaloparatide and romosozumab—have been approved to be applied in the pharmacotherapy of osteoporosis. Abaloparatide is an analog of the first 34 amino acids of human parathyroid hormone-related peptide [hPTHrP(1–34)], in which 20 amino acids are changed compared with their natural counterparts. Romosozumab is a humanized monoclonal antibody to sclerostin. Odanacatib is a new research molecule that has demonstrated cathepsin K inhibition and significant antifracture efficacy, but phase 3 clinical trials of odanacatib were stopped due to an increased risk of stroke [1,2].

Teeth are special types of bone constantly exposed to harmful agents, which can lead to dental caries or periodontitis [1,5]. The World Health Organization (WHO) estimates that approximately 3.5 billion people are affected by oral diseases [6]. According to a Centers for Disease Control and Prevention (CDC) report, in the USA, the prevalence of periodontitis in adults is 47.2% in those aged 30 years and above [7]. Frencken, Jo E., et al. have reported a decrease in the frequency of caries, but a high prevalence of periodontitis is observed worldwide [8]. A few studies have related the recovery of the dental pulp and bone loss in periodontitis to adenosine-5′-triphosphate (ATP) signaling [9,10]. Scientific evidence suggests that the activation of P2X7R via the ATP pathway may influence the regulation of osteoblasts and their activities and may be associated with bone resorption and formation [11,12].

To date, only one review has focused on the effects of selected mushrooms as potential agents in the prevention or treatment of osteoporosis [13]. In addition, some studies have provided information related to the impact of *Cordyceps militaris* or cordycepin on skeletal system diseases [14,15].

**Figure 1:**
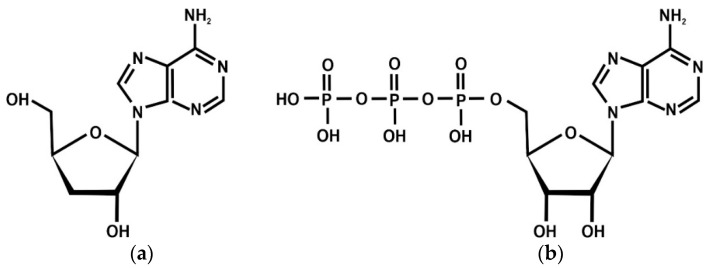
(**a**) Chemical structure of cordycepin; (**b**) Chemical structure of adenosine-5′-triphosphate.

The entomopathogenic fungi *Cordyceps* spp. (classified to the *Ascomycota* group) have traditionally been used in ethnomedicine in Asian countries such as China, Japan, Korea, and India. These species are unique parasites of larvae of selected species of moths. Various bioactive constituents such as cordycepin, ergothioneine, γ-aminobutyric acid, lovastatin, and cordymin have been isolated from the fruiting bodies of *C. militaris*, along with new compounds such as cordyxanthins and cordyrroles. Cordycepin (3′-deoxyadenosine) is a nucleoside, which is a structural analog of adenosine (Figure 1).

Cordycepin is a natural biometabolite produced by *Cordyceps* spp., which was first isolated from fruiting bodies of *C. militaris* (caterpillar mushroom). Animal experiments showed that *Cordyceps* spp. and cordycepin interact via the adenosine receptors (ARs), AMPK, and the ATP signaling pathway. The chemical structure of ATP is presented in Figure 1 [16].

Experiments on pigs demonstrated the immunostimulatory and antioxidant activities of *C. militaris*, as well as the ability to improve growth performance [17,18].

Most of the studies involving humans were conducted using *Cordyceps sinensis*, which is an edible mushroom that has a long history of consumption and is used as a food and dietary supplement in the EU (before 1997). In addition, *C. sinensis* as an ingredient has been authorized by the European Food Safety Authority (EFSA) and European Commission (EC). In contrast, *C. militaris* is classified not as an edible mushroom but as a medicinal mushroom. However, it is not an authorized ingredient for food and dietary supplements in the EU. This novel food cannot be used for human consumption without approval from the EC and EFSA. *C. sinensis* is used in Chinese pharmacopoeia, and its adenosine concentration is considered the primary quality indicator [19,20,21]. Although various biological activities of *Cordyceps* spp. and cordycepin have been reported, their effects on bone functions have not been investigated adequately [15,22,23]. In recent years, the effects of *Cordyceps* spp. and cordycepin on bone tissue or dental pulp stem cells have gained attention because the human skeletal system also includes teeth, a unique type of bone. Therefore, this review also included the keywords “teeth” or “dental” in the queries. This review aimed to collect and analyze the data available regarding the influence of *Cordyceps* spp. or cordycepin on the functions of bones and teeth and associated processes. To the best of our knowledge, this is the first systematic review of this issue.

## 2. Results

Studies performed on cells and animal models have shown the benefits of using *Cordyceps* spp. and/or cordycepin as a potential intervention in the treatment of selected bone diseases. All data are presented in Table 1.

In vitro experiments confirmed that 100, 150, and 200 µg/mL concentrations of *C. sinensis* increased the number of erythroid cells: both colony-forming unit—erythroid (CFU-E) and burst-forming units—erythroid (BFU-E). A high dose of *C. sinensis* (250 µg/mL) suppressed the proliferation of CFU-E and BFU-E. An in vivo experiment showed that 100, 150, and 200 mg/kg/day doses of *C. sinensis* stimulated erythropoiesis and increased the number of CFU-E and BFU-E in mouse bone marrow. The mushroom used in this experiment did not contain cordycepin, and only included d-mannitol, polysaccharides, and amino acids, whose exact contents are not known [24]. In vitro tests demonstrated that the water extract of *C. militaris* affected the maturation of dendritic cells in the murine bone marrow. The *C. militaris* water extract was not cytotoxic for murine bone marrow cells up to the concentration of 250 µg/mL. However, the exact content of cordycepin or other active ingredients was not determined in this experiment [25].

An in vitro test showed that the intervention of *C. sinensis* stimulated the proliferation and differentiation of bone marrow cells. In vivo experiments on mice demonstrated that the *C. sinensis* water extract (dose 50 mg/kg/day) prevented the death of bone marrow stem cells due to total body irradiation (TBI) at lower doses in the range of 5.5 and 6.5 Gy. *C. sinensis* improved the survival of mice administered lethal TBI [26]. An in vitro experiment showed that the *C. sinensis* water extract (in dose-dependent concentrations of 10, 50, and 100 µg/mL) inhibited the Receptor Activator for Nuclear Factor κB Ligand (RANKL)-induced osteoclast differentiation in mouse bone marrow cells and the monocyte/macrophage cell line RAW264.7. Up to the concentration of 100 µg/mL, the *C. sinensis* water extract did not show any cytotoxic effect in RAW264.7 cells. However, information on active ingredients was not provided in these works [27].

In mice with leukopenia induced by paclitaxel, oral administration of the *C. sinensis* water extract (50 mg/kg/day) for 3 weeks affected osteoblast differentiation, which was correlated with the protection of bone marrow stem cells [28].

In ovariectomized (OVX) osteopenic rats, the administration of *C. sinensis*, which is rich in strontium, resulted in a decrease in bone resorption, a decrease in urinary calcium excretion, an improvement in bone formation, and an increase in the mineral content of bones [29].

*C. sinensis* rich in strontium ranelate reduced bone loss in experimental OVX osteopenic rats, in which a decrease in alkaline phosphatase (ALP) and tartrate-resistant acid phosphatase (TRAP) activities, a decrease in the interferon gamma (IFN-γ) level, and an increase in the osteocalcin (OC) and estradiol levels in plasma were observed [30].

In rats, oral administration of 300 or 500 mg/kg/day of *C. sinensis* water extract (cordycepin 5.27 µg/g, adenine 306.27 µg/g, adenosine 212.74 µg/g, ergosterin 340.81 mg/g, among others) for eight weeks was shown to increase the bone mineral density and prevent disuse-induced bone loss and deterioration of trabecular microarchitecture. Compared with the control group, a decrease in the total serum calcium concentration was observed in all groups treated with the *C. sinensis* water extract. However, only a high dose (500 mg/kg/day) of the *C. sinensis* water extract significantly decreased the level of urinary calcium excretion (47.35–48.52%). Furthermore, 500 mg/kg/day of the *C. sinensis* water extract resulted in an increased OC level, but decreased serum levels of ALP, TRAP, crosslinked carboxyterminal telopeptide of type I collagen (CTX), and IFN-γ. Among all the included studies, only one study is a randomized controlled trial [31].

Two studies related to queries with *C. sinensis* active ingredients other than cordycepin, namely cordymin and isoflavones, were included in this review [32,33].

In broiler chicken, the feeding of 2 g of fermentation products of *C. militaris* per 1 kg of feed contributed to an increased calcium content in the tibia compared with the control group. The concentration of cordycepin was determined to be 5.09 mg/g of fermentation products of *C. militaris* [34].

In an inflammatory-induced osteoporosis model of experimental rats, oral administration of 20 mg/kg cordycepin showed anti-inflammatory activity and limited bone loss, which in turn decreased the levels of CTX, maleic dialdehyde, myeloperoxidase, IL-1β, and TNF-α in the serum and increased the level of OC [35].

In in vitro experiments, cordycepin showed a dose-dependent regulation of osteogenesis of human adipose-derived mesenchymal stem cells. A low concentration of cordycepin (10 µg/mL) promoted osteogenic differentiation, whereas a higher concentration (40 µg/mL) inhibited it. However, a moderate concentration of cordycepin (20 µg/mL) did not affect osteogenic differentiation. This confirms that cordycepin prevents the TNF-α-induced inhibition of osteogenic differentiation and that 10 µg/mL of cordycepin suppresses TNF-α-activated NF-κB signaling via the inhibition of IκBα phosphorylation. It should also be emphasized that a high concentration of cordycepin (20–40 µg/mL) can induce cell death [36].

An in vitro test demonstrated that 50 µg/mL of cordycepin (isolated from *C. militaris*) decreased the ALP and TRAP activities. In vivo experiments on rodents showed that oral administration of cordycepin can prevent bone loss and improve the mechanical strength of bones (the femoral neck) in an experimental model of osteoporosis. A 20 mg dose of cordycepin was reported to decrease the CTX level in serum, decrease the ALP and TRAP activities, and increase the OC level in plasma in OVX osteopenic rats [37].

**Figure 2:**
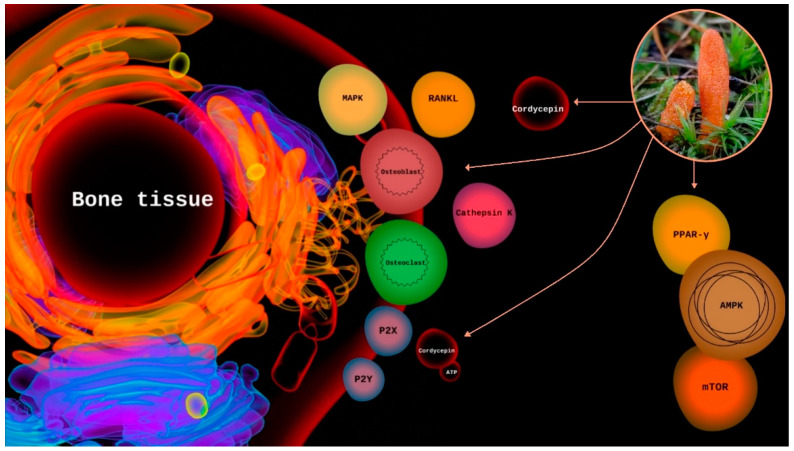
The mechanism of actions—influence on bone tissue (illustrative image).

In vitro and in vivo experiments showed that cordycepin induced osteogenic differentiation of bone marrow mesenchymal stromal cells (BMMSCs) by limiting oxidative stress in OVX mouse and related mouse models [38]. In addition, in vitro tests showed that cordycepin and the water extract of *C. militaris* (6.8% cordycepin content) inhibited osteoclast differentiation. Cordycepin was more effective in inhibiting osteoclast formation than the *C. militaris* water extract (Figure 2). 

The biological activity of these two investigated materials was found to be associated with the inhibition of RANKL, but cordycepin was more effective in inhibiting RANKL-induced osteoclastogenesis than the *C. militaris* water extract. In addition, *C. militaris* and cordycepin inhibited the mRNA expression of genes related to osteoclastogenesis such as cathepsin K, TRAP, matrix metalloproteinase-9 (MMP-9), and the nuclear factor of activated T-cells, cytoplasmic 1 (NFATc1). Furthermore, cordycepin significantly suppressed RANKL-induced p38 and NF-κB phosphorylation. Moreover, in vivo experiments on a mouse model of lipopolysaccharide-mediated bone loss showed that oral administration of 100 µg/g of the *C. militaris* water extract for 8 days prevented bone loss in the range of 20–40% [39]. In vitro tests showed that cordycepin inhibited RANKL-induced osteoclastogenesis. Cordycepin increased the expression of IRF-8 but suppressed the expression of NFATc1. In vivo experiments indicated that 4 weeks of oral administration of 10 mg/kg/day of cordycepin in OVX mice prevented bone loss, rescued bone microarchitecture, and restored bone mineralization [40]. Furthermore, in vitro tests using human bone mesenchymal stem cells and animal experiments on rats demonstrated the osteoprotective effect of cordycepin [41].

Results of in vitro tests showed that cordycepin enhanced osteoblast differentiation and matrix mineralization, which was related to the induction of markers such as BMP2, RUNX2, and the osterix (OSX) signaling pathways. Cordycepin in a concentration of 1 µM suppressed osteoclastogenesis by inhibiting the RANKL, TNF-receptor associated factor 6 (TRAF6), and the mitogen-activated kinase (MAPK) pathway. This activity was related to the suppression of gene expression of cFOS, cathepsin K, and NFATc1 [42]. In vitro tests showed that 10 µg/mL of cordycepin promoted osteogenesis of bone marrow mesenchymal stem cells. An in vivo experiment demonstrated that a 10 mg/kg/day dose of cordycepin promoted and accelerated fracture healing via hypoxia in a rat model of a closed femoral fracture. Nevertheless, these studies have some limitations, such as the lack of histological examination in experimental animals. The results of in vivo experiments were based on radiographic evaluations and the 4-point bending mechanical test [43]. An in vitro experiment and an in vivo test on mice confirmed that cordycepin induced apoptosis and inhibited the proliferation of osteosarcoma cells. In vitro tests showed that cordycepin decreased the expression of markers Bcl-2, MMP-2, and MMP-9 but increased the expression of PARP and proapoptotic protein Bax. Cordycepin activated the AMPK pathway and suppressed the AKT/mTOR signaling pathway, which inhibited the growth of osteosarcoma cells. In an in vitro experiment, a combination of cordycepin and cisplatin showed a stronger antitumor effect and a higher decrease in colony formation and cell invasion abilities than either drug used individually. Furthermore, in an in vivo experiment, a combination of cordycepin and cisplatin synergistically inhibited the growth and invasion of osteosarcoma cells, thus reducing the tumor volume. Thus, the coadministration of these two compounds showed a significantly stronger inhibitory effect on tumor growth than either drug used individually [44].

Cordycepin application resulted in adipogenic and osteogenic changes in dental pulp stem cells isolated from healthy tooth extracts. Cordycepin in a concentration of 5 µM significantly decreased the expression of genes related to adipogenesis, such as peroxisome proliferator-activated receptor gamma (PPARγ), fatty acid-binding protein 4 (FABP4), lipoprotein lipase (LPL), and CCAAT/enhancer-binding protein alpha (C/EBPα). With regard to osteogenesis, cordycepin was shown to increase the expression of runt-related transcription factor 2 (RUNX2), type I collagen alpha 1 (COL1A1), transcription factor Sp7/Osterix (OSX), and bone gamma carboxyglutamic acid-containing protein/osteocalcin (OCN) genes [45].

Cordycepin administration influenced the migratory ability of dental pulp stem cells. The administration of a higher concentration (5 µM) of cordycepin significantly decreased the level of reactive oxygen species (ROS), decreased PPARγ expression, but increased RUNX2 expression as the osteogenesis regulator gene, thus increasing the migration of dental pulp stem cells. Cordycepin at 1, 2.5, and 5 µM doses increased the number of stem cells. Cordycepin did not affect the viability of dental pulp stem cells up to the concentration of 5 µM; however, higher concentrations (above 10 µM) of cordycepin resulted in cytotoxic effects [46].

A study reported that the nucleosides and amino acids isolated from *C. sinensis* attenuate the myelosuppression induced by cyclophosphamide in experimental mice [47].

Bearing in mind the limited amount of research on the entire issue, it was decided to briefly characterize each study. According to Weibiao P. et al., the extract of *C. sinensis* contributes to inhibited RANKL-induced osteoclastogenesis, similar to the MAPK pathway in in vitro tests performed on bone marrow macrophages (BMMs). An in vivo experiment showed that administration of the *C. sinensis* extract for 6 weeks prevented OVX-induced osteoporosis in mice, retained the bone volume by attenuating osteoclast activity, decreased the TRAP concentration, and increased the OC level in serum signed as bone gla-protein (BGP). However this work was not written in the English language, an extensive discussion of this publication was unable due to language barrier [48].

## 3. Discussion

### 3.1. Ergothioneine

Ergothioneine (2-thiol-l-histidine-betaine), also known as thioneine, is a sulfuric derivative of l-histidine with betaine attached to it. *C. militaris* is a source of ergothioneine, which is present in high concentrations in mycelial cultures at 10.4 mg/100 g dry weight (dw), as well as in self-cultivated and commercially available fruiting bodies [16].

Cohen, Nachshol et al. reported the highest content of ergothioneine in fruiting bodies in the range of 41.0 mg to 100 g dw [49]. The EFSA assessed the safety margins of synthetic l-ergothioneine (under the trade name Ergoneine) in the concentration of 470 mg/kg body weight (bw) per day for adults, except for pregnant and breastfeeding women [50]. The human body cannot biosynthesize ergothioneine. In recent studies, the specific ergothioneine transporter (ETT) has been identified, and a high concentration of ergothioneine has been confirmed in some tissues and cells, such as erythrocytes, the spleen, liver, and eyes [51,52].

In healthy subjects, daily supplementation of 5 or 25 mg of ergothioneine contributed to only a slight decrease in the level of ROS markers. Perhaps the antioxidant activity of ergothioneine may be more significant in specific cases as a higher production of ROS was observed in diseases with an inflammatory response or intense physical effort [53]. In a study of 3236 participants, Smith, Einar, et al. reported that the high level of ergothioneine can be an indicator of a lower risk of cardiometabolic disease and lower mortality [54]. In humans, the level of ergothioneine decreases with age and thus can be correlated with the onset of some diseases in the elderly after 60 years of age [52,55].

One of the highest concentrations of ergothioneine was observed in bone marrow, which was reported in the early 1950s [56]. Another study confirmed that the expression of human ETT is a strong signal from the blood cells and bone marrow [57].

### 3.2. Purinergic Signaling

The impact of ARs on purinergic signaling may contribute to the regulation of proliferation, differentiation of dental pulp stem cells and bone marrow, and the modulation of osteogenesis and bone repair. An in vitro test showed that the selective stimulation of A_1_R contributes to a higher differentiation of dental pulp stem cells into osteoblasts. The selected agonist of A_1_R was related to the activation of the phosphatidylinositol 3-kinase (PI3K)/Akt and MAPK pathways [58]. ATP can activate purinergic receptors through the metabotropic receptor family (P2Y) and the ionotropic receptor family (P2X). The P2X7R subtype has a significant positive influence on osteogenesis. The influence of the P2X7R expression on BMMSCs is correlated with the regulation of the balance between apoptosis and the formation of osteoblasts and osteoclasts. The P2X7R subtype has a positive influence on shockwave-induced osteogenesis in human MSCs and osteogenic differentiation of MSCs in a mouse model of osteoporosis. Some studies indicated that the mTORC2 pathway is also involved in bone homeostasis [59,60].

Kim, Jaeyoon, et al. reported that adenosine and cordycepin stimulate the Wnt/β-Catenin signaling pathway by activating the subtypes adenosine A_2A_ and A_2B_ receptors, promoting tissue remodeling and stimulating the production of growth factors correlated with tissue repair [61].

Dentin is generated by odontoblasts—cells contained in the pulp inside the dentin, including the nerve. Purinergic receptors are also present in human dental pulp cells (HDPCs). These cells express selected subtypes of P2X and all subtypes of P2Y receptors. Interestingly, stimulation of these cells with a low ATP concentration (10 µM) enhanced HDPC proliferation, whereas higher doses (800 µM) arrested cell proliferation while inducing odontoblast differentiation, which was associated with ERK/MAPK activation. A number of studies have reported the ability of HDPCs to differentiate into odontoblasts and osteoblasts. The P2X7R subtype was detected in odontoblasts in the dental pulp. Odontoblasts can release ATP, which interacts with axons in the pulp via P2X3R [9,62]. Another study on human bone samples (from explants) demonstrated that P2X7R is upregulated by NFATc1 [63].

### 3.3. Cordyceps *spp.* as a Source of Calcium, Phosphorus, Fluoride, and Vitamin D

In the European Union (EU), health claims are authorized and approved by the EFSA and the EC, which state that calcium, phosphorus, and vitamin D contribute to the maintenance of normal functions of bones and teeth. Vitamin D also contributes to the normal absorption and utilization of calcium and phosphorus. Fluoride contributes to teeth mineralization [19]. The adequate supplementation of calcium and vitamin D plays a key role in the development of the skeletal system and the prevention of bone atrophy, whereas their deficiency causes rickets in childhood—which is a period of intensive growth—and leads to low maximum bone mass, bone atrophy, and osteoporosis. Calcium is part of the bone matrix, which strengthens bones and is stored in them. When necessary, the body acquires the stored calcium, which is triggered by the increased synthesis and secretion of the parathyroid hormone from the parathyroid glands. Calcium is supplemented to the body via the diet. The daily calcium requirement varies with age. On average, the daily requirement of calcium is estimated to be 1000 mg, but, for example, during rapid growth or lactation, it increases to 1500–2000 mg. In addition, an increased supply of calcium is necessary for postmenopausal women (about 1500 mg). The rate of bone atrophy—determined during this period by the decrease in the estrogen level in a woman’s body—is largely dependent on proper calcium supplementation. Dietary calcium deficiency is one of the factors involved in the destruction of bone tissue to use this element for the requirements of other organs [64]. In turn, vitamin D in its active form (1,25–OH cholecalciferol) as the calcitropic hormone affects calcium absorption, body calcium–phosphorus homeostasis, and mineralization of the bone matrix. In adults, the appropriate dose of vitamin D, including both the vitamin produced in the body and the vitamin consumed in foods, is approximately 4000 IU. In medical recommendations for daily vitamin D requirements, it is important to take into account factors such as climate zone [64]. Nutrient reference values (NRVs) or daily reference intakes of calcium and vitamin D in adults are estimated to be 1000–1300 mg and 5 µg, respectively. Tolerable upper intake levels (ULs) of calcium and vitamin D are estimated to be 2500 mg/day and 100 µg/day, respectively [19,65].

In the human body, phosphorus plays a number of important functions and is essential for its proper functioning. Similar to calcium, phosphorus is also a building block and is involved in the mineralization of bones and teeth. Its content in the human body is approximately 1% of body weight (bw). More than 80% of phosphorus is present in teeth and bones. The presence of phosphate in the blood is attributable to absorption from the gastrointestinal tract, excretion by the kidneys, and bone metabolism. The level of phosphorus in the human body is regulated by parathormone and vitamin D [64]. In adults, the NRV of phosphorus is estimated to be 700 mg; however, adequate data are not available to derive its UL [19,65].

Fluoride plays many important functions in the human body. It is essential for the proper development of bones and teeth, stimulates the formation of new bone tissue, and participates in the conversion of calcium phosphate to apatite—the major mineral component of bones. It increases the mineralization of tooth tissue, reduces the growth of caries-forming bacteria, promotes remineralization, and increases the resistance of teeth to the acidic environment in the mouth, thus preventing caries. In children, fluoride is primarily involved in the development of bones and teeth. However, in adults, it is necessary for bone remodeling. The fluoride content of the human body is estimated to be approximately 3 mg/kg of bw. Fluoride requirements depend on age, gender, and weight [64]. In adults, the NRV of fluoride is estimated to be 3.5 mg. Adequate intake (AI) of fluoride is estimated to be 0.05 mg/kg bw per day, which indicates the maximum daily intake of 3.5 mg for individuals with a weight of 70 kg. The UL of fluoride is estimated to be 7 mg/day [19,65,66].

Mushrooms contain many essential minerals, such as elements important in the development of bones and teeth. Hence, their natural sources are constantly being sought. Mushrooms are rich natural sources of many bioelements. High concentrations of these bioelements in different species of the genus *Cordyceps* make them alternative sources of these minerals in the human diet. For example, the results of the macroelement composition analysis in fruiting bodies and mycelial biomass of mycelial cultures of *C. militaris* showed the significant presence of calcium and phosphorus as follows: calcium—797 mg/kg (fruiting bodies) and 11 mg/kg (biomass from mycelial cultures) and phosphorus—7196 mg/kg (fruiting bodies) and 14.293 mg/kg (biomass from mycelial cultures) [67].

Another study evaluated the nutritional and health-promoting effects of three edible and medicinal mushrooms of the *Cordyceps* genus: *Cordyceps gunnii*, *Cordyceps jiangxiensis*, and *Cordyceps taii*. The fruiting bodies of these three species were found to be significant sources of calcium and vitamin D. The calcium content was 1460 mg/100 g dw, 3850 mg/100 g dw, and 5410 mg/100 g dw and the vitamin D content was 0.25 mg/100 g dw, 0.23 mg/100 g dw, and 0.34 mg/100 g dw in *C. gunnii*, *C. jiangxiensis*, and *C. taii*, respectively. In this study, *C. taii* showed the highest level of calcium, which was significantly different from those of the other two species. Furthermore, all *Cordyceps* species showed considerably higher levels of calcium than *C. sinensis* and many wild mushroom species (0.17–500 mg/kg) [68,69]. The fruits of *C. militaris* enriched with selenium are industrially cultivated as functional or medicinal foods in China and Southeast Asia. The presence of phosphorus in these fruiting bodies was confirmed using synchrotron-based X-ray absorption spectroscopy at the 20-ID beamline (PNC/XOR) of APS [70]. The fluoride content of various Japanese and Chinese medicinal raw materials was determined using alizarin complex spectrometry. In dried fruiting bodies of *C. sinensis*, the fluoride content was estimated to be 23.86 ppm [71]. Other studies have reported that fluoride contributes to biomass growth and increases the bioactivity, including the antioxidant and anticancer activities of mycelial culture extracts [72].

The present study, published in 2022, was aimed to determine the content of biologically active substances, i.e., bioelements, in fruiting bodies (commercially available and self-cultivated), the mycelium, and two dietary supplements containing *C. militaris*. In addition, the calcium content of extracts from digestive juices was estimated in an artificial digestive tract model after extraction from digestive juices in an artificial digestive tract model was estimated, among other things. Furthermore, the content of potentially bioavailable substances in the human body was estimated. The calcium content in both mycelium cultures (351 mg/100 g dw) and fruiting bodies (312 mg/100 g dw) far exceeded the previously determined amounts. To determine the bioavailability of bioelements and organic compounds to the human body, a specially designed Gastroel-2014 apparatus was used to analyze the release of mycelium (from in vitro cultures, fruiting bodies, and commercially available dietary supplements) into artificial gastric and intestinal juices. The bioavailability of these substances in the mycelium was evaluated by determining the concentration of substances in the extracts of digestive juices (artificial gastric juice at 60 min and artificial intestinal juice at 150 min). The contents of the analyzed bioelements in the starting material were found to be significantly higher than the potentially bioavailable content, i.e., determined after in vitro simulated digestion. Compared with the intestinal juice, a higher content of bioelements, including calcium, was observed in the gastric juice, which was higher than those reported in previous studies. The richest sources of bioavailable calcium were analyzed in the extract of the artificial gastric juice. In addition, in the intestinal juice, the highest content of calcium was observed in commercially available dietary supplements. These results highlight the need for further research into the bioavailability of *C. militaris* bioelements [16].

## 4. Materials and Methods

**Figure 3:**
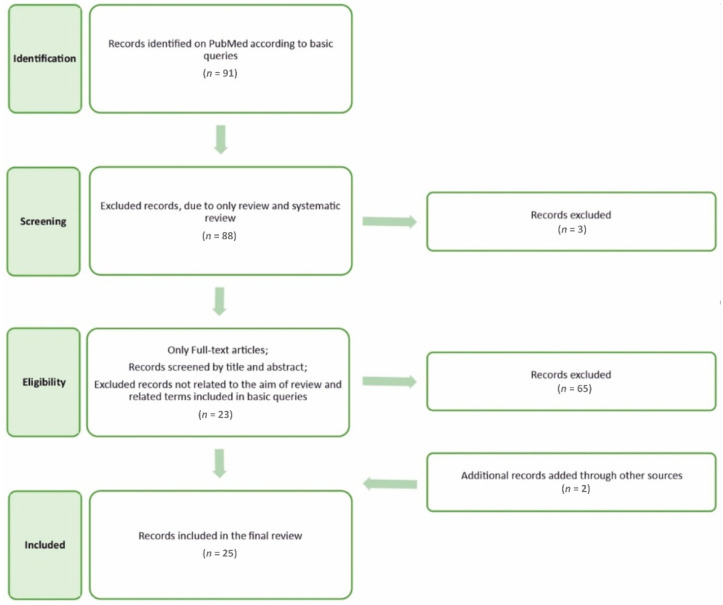
Flowchart of the methodology and data workflow.

Data were collected using PubMed searches from 16 September 2022, to 16 October 2022. The following queries were used: “((cordyceps) OR (cordycepin)) AND ((bone) OR (osteoporosis) OR (dental) OR (teeth) OR (mineralization))”. The first query provided 91 records, of which three were excluded as they were reviews or systematic reviews. Only full-text articles were considered eligible, and in the next stage, all records were screened by their title and abstract. A further 65 records that were not related to the aim of the review and queries were excluded, whereas two records were added from other sources. Hence, this review included 25 records. The methodology and data workflow are presented in Figure 3.

## 5. Conclusions

Many studies have focused on the potential treatment of osteoporosis or the antitumor effect on bone tissue. In recent years, scientific evidence on the application of cordycepin from dental pulp stem cells in stomatology has been increasing, but only an in vitro experiments. The influence of ARs on the purinergic signaling pathway and their impact on ATP are not fully understood. These potential signaling pathways may contribute to the regulation of proliferation and the differentiation of stem cells, the regulation of osteogenesis, and improvements in bone repair and rebuilding. These pathways are crucial to the development of regenerative medicines and new methods of treatment or interventions in recovery after traumatic injuries, convalescence, and terminal-stage or devastating diseases. This will provide a direction for further research in this area, not necessarily on mushroom raw material or even the isolated parent compound cordycepin, but on new molecules that are structural design project analogs of nucleosides, such as those from *C. militaris* mushrooms.

## Figures and Tables

**Table 1:** Studies included in this review and their details.

Type of Study	Investigated Subject	Investigated Material	Dose/Concentration	Results/Outcome	Comment	References
**In vitro**	CFU-E; BFU-E	*C. sinensis*	100, 150, 200 µg/mL	Increases the number of erythroid cells CFU-E and BFU-E	Content of cordycepin not determined	[24]
**In vivo**	Mice	*C. sinensis*	50, 100, 150, 200, 250, 300 mg/kg/day; intraperitoneal injection for 5 days	Stimulates erythropoiesis; increases the number of CFU-E and BFU-E in mouse bone marrow	Content of cordycepin not determined	
**In vitro**	BMCs	*C. militaris* water extract	Up to 250 µg/mL	Induced maturation of murine BMCs	Content of cordycepin not determined	[25]
**In vitro**	BMCs: BMMSCs; BMHSCs	*C. sinensis* water extract	500 µg/mL	Stimulate the proliferation of BMCs; protection of BMCs from radiation cytotoxicity	Content of cordycepin not determined	[26]
**In vivo**	Mice	*C. sinensis* water extract	50 mg/kg/day; oral administration for 1 week	Protection of mice bone marrow after TBI; increase a survival of mice receiving TBI	Content of cordycepin not determined	
**In vitro**	Mouse BMCs and monocyte macrophage RAW 264.7 cells	*C. sinensis* water extract	10, 50, 100 µg/mL	Inhibition of RANKL-induced osteoclast differentiation via NF-κB pathway	Content of cordycepin not determined	[27]
**In vitro**	BMMSCs; BMHSCs	*C. sinensis* extract	500 µg/mL	Stimulate the differentiation of BMCs	Content of cordycepin not determined	[28]
**In vivo**	Mice with leukopenia induced by paclitaxel	*C. sinensis* extract	50 mg/kg/day for 3 weeks	Enhancement recovery of mice from leukopenia induced by paclitaxel	Content of cordycepin not determined	
**In vivo**	OVX osteopenic rats	*C. sinensis* rich in strontium	10 mL a mixture of *C. sinensis* rich in strontium; oral administration for 8 weeks	Decreases bone resorption, increases bone formation; increase the estradiol level	Content of cordycepin not determined	[29]
**In vivo**	OVX osteopenic rats	*C. sinensis* rich in strontium	10 mL a mixture of *C. sinensis* rich in strontium; oral administration for 8 weeks	Decrease of ALP and TRAP activity; decrease of CTX and IFN-γ level; increase the OC and estradiol level	Content of cordycepin not determined	[30]
**In vivo**	Rats	*C. sinensis* extract	100, 300, 500 mg/kg/day; oral administration for 8 weeks	Increase the bone mineral density; prevent disuse-induced bone loss	Content of cordycepin 5.27 µg/g	[31]
**In vivo**	Model diabetic osteopenic rats	Cordymin from *C. sinensis*	20, 50, 100 mg/kg/day; intraperitoneal injection for 5 weeks	Decrease of ALP and TRAP activity	—	[32]
**In vivo**	OVX rats	Isoflavones from *C. sinensis*	20, 50, 100 mg; oral administration for 8 weeks	Prevent of bone loss induced by estrogen deficiency; decrease of urinary Ca excretion; decrease of ALP and TRAP activity; decrease of CTX and IFN-γ level; increase the OC and estradiol level	Performed a histological examination of bone	[33]
**In vivo**	Broiler chicken	Fermentation products of *C. militaris*	1–4 g fermentation products of *C. militaris* per 1 kg of feed	Increased calcium content in tibia	Content of cordycepin 5.09 mg/g. High dose at 4 g/kg of fermentation products of *C. militaris* had a negative impact on bone mineralization	[34]
**In vivo**	Rats with IMO	Cordycepin	5, 10, 20 mg/kg; oral administration two times daily for 3 weeks	Prevent of bone loss; decrease of CTX, MDA, IL-1β, TNF-α level in the serum; increase the OC level	Performed a histological examination of liver, not bone	[35]
**In vitro**	ADMSCs	Cordycepin	10–40 µg/mL	Low concentration of cordycepin 10 µg/mL promoted osteogenic differentiation	High concentration of cordycepin 20–40 µg/mL induce cell death	[36]
**In vitro**	Murine mesenchymal stem cells	Cordycepin	10, 20, 50 µg/mL	Decrease of ALP and TRAP activity	—	[37]
**In vivo**	OVX osteopenic rats	Cordycepin	5, 10, 20 mg/kg; oral administration two times daily for 3 weeks	Prevention of bone loss; increase the OC level; decrease the CTX level	Performed a histological examination of bone	
**In vitro**	BMMSCs	Cordycepin from *C. militaris*	0.1, 0.2, 0.5, 1, 2 mM; 1, 5, 10, 20, 40, 80 μg/mL	Promoted osteogenic differentiation; decrease of ALP and TRAP activity	—	[38]
**In vivo**	OVX mice	Cordycepin from *C. militaris*	1, 5, 10 and 20 mg/kg; intraperitoneal injection for 8 weeks	Increase of calcium content	Did not perform a histological examination of bone	
**In vitro**	Mouse monocyte macrophage RAW 264.7 cells	*C. militaris* and cordycepin	1, 10 µg/mL	Inhibition of osteoclast differentiation	—	[39]
**In vivo**	Mouse model of lipopolysaccharide-mediated bone loss	*C. militaris* (content cordycepin)	100 µg/g; oral administration for 8 days	Prevention of bone loss	Did not perform a histological examination of bone; performed a micro-CT analysis	
**In vitro**	Mouse monocyte macrophage RAW 264.7 cells and BMMs	Cordycepin	0.01, 0.05, 0.1, 0.5, 1, 5, 10 μg/mL	Inhibition of RANKL-induced osteoclastogenesis	Inhbitory effects of cordycepin started at 0.1 μg/mL; concentration of cordycepin above to 5 μg/mL; cytotoxic effect started at concentration of 5 µg/mL	[40]
**In vivo**	OVX mice	Cordycepin	10 mg/kg/day; Oral administration for 4 weeks	Prevention of bone loss	Performed a histological analysis; performed a BMD analysis	
**In vitro**	HBMSCs	Cordycepin	0.1, 1, 10 μg/mL	Decrease of ALP activity	Even a high concentration at 10 μg/mL not suppress a proliferation of HBMSCs	[41]
**In vivo**	Rats model of alcohol-induced ONFH	Cordycepin	10 mg/kg/day; intraperitoneal injection for 6 weeks	Cordycepin prevent on alcohol-induced ONFH	Performed a micro-CT analysis	
**In vitro**	Murine MC3T3-E1 and RAW264.7 cells	Cordycepin	0.5, 1 µM	Inhibition of RANKL-induced osteoclast differentiation	Even a high concentration at 5 µM provide no cytotoxic effect	[42]
**In vitro**	BMMSCs	Cordycepin	10 µg/mL	Promotes osteogenesis	—	[43]
**In vivo**	Rat model of closed femur fracture	Cordycepin	10 mg/kg/day	Accelerate a fracture healing	Performed a histological analysis	
**In vitro**	Osteosarcoma cells and osteoblast cells	Cordycepin	100, 200, and 400 µM	Inhibition of proliferation osteosarcoma cells; induce of osteosarcoma cell apoptosis	—	[44]
**In vivo**	Mice	Cordycepin	40 mg/kg/day; intraperitoneal injection for 32 days	Inhibited osteosarcoma cell invasion	—	
**In vitro**	Dental pulp stem cells	Cordycepin	0.5, 1, 2.5, 5, 10, 25, 50 µM	Increased the expression of RUNX2, COL1A1, OSX, OCN	—	[45]
**In vitro**	Dental pulp stem cells	Cordycepin	1, 2.5, 5, 10, 25, 50 µM	Increased the migration of dental pulp stem; increase the number of stem cells	Concentration above at 10 µM resulted an cytotoxic effects	[46]
**In vivo**	Mice with myelosuppression induced by cyclophosphamide	Nucleosides and amino acids from *C. sinensis* (natural and artificially-cultivated)	0.48–1.78 (contents ratio of artificially-cultivated versus natural *C. sinensis*)	Protection against myelosuppression induced by cyclophosphamide	Content of cordycepin not determined	[47]

Colony-forming unit—erythroid (CFU-E); Burst-forming unit—Erythroid (BFU-E); Murine bone marrow cells (BMCs); Bone marrow mesenchymal stem cells (BMMSCs); Bone marrow hematopoietic stem cells (BMHSCs); Total body irradiation (TBI); Receptor Activator for Nuclear Factor κB Ligand (RANKL); Nuclear factor kappa-light-chain-enhancer of activated B cells (NF-κB); Ovariectomized (OVX); Alkaline phosphatase (ALP); Tartrate-resistant acid phosphatase (TRAP); Cross-linked carboxy terminal telopeptide of type I collagen (CTX); Interferon gamma (IFN-γ); Osteocalcin (OC); Inflammation-induced osteoporosis (IMO); Malondialdehyde (MDA); Interleukin 1 beta (IL-1β); Tumor necrosis factor alpha (TNF-α); Human adipose-derived mesenchymal stem cells (ADMSCs); Mesenchymal Stem Cells (MSCs); Bone marrow macrophages (BMMs); Human bone mesenchymal stem cells (HBMSCs); Bone mineral density (BMD); Osteonecrosis of the femoral head (ONFH); Runt-related transcription factor 2 (RUNX2); Type I collagen alpha 1 (COL1A1); Osterix (OSX); Bone gamma carboxyglutamic acid-containing protein/osteocalcin gens (OCN).

## Data Availability

The data are contained within this article.
